# Analysis of differentially expressed genes in cold-exposed mice to investigate the potential causes of cold-induced hypertension

**DOI:** 10.3892/etm.2014.1703

**Published:** 2014-05-08

**Authors:** BUXIONG TUO, CHAOMIN LI, LIJING PENG, MINGXIA YE, WEI LIU, XIAOLAN ZHONG, HUI LI

**Affiliations:** Department of Cardiology, 451 Hospital of PLA, Xi’an, Shaanxi 710054, P.R. China

**Keywords:** cold exposure, hypoxia, oxidative stress, hypertension, gene expression, protein-protein interaction network

## Abstract

Cold exposure is considered to be an important contributing factor to the high morbidity of hypertension. In order to elucidate the cause and mechanism of cold-induced hypertension (CIH), gene expression analysis was performed on microarray data for two groups of cold-exposed mice (4°C for 1 week and 4°C for 5 weeks, three replicates per group) and their respective control groups maintained at 30°C. Analysis results indicated that the differentially expressed genes with the most significance were associated with adaptive thermogenesis, fatty acid metabolism and energy metabolism. The expected marked increase in metabolism during cold exposure caused tissue hypoxia. Genes involved in the hypoxia-inducible factor signaling pathway were activated. In addition, genes associated with oxidative stress were significantly upregulated, including superoxide dismutase 2 (*SOD2*) and epoxide hydrolase 2 (*EPHX2*). The majority of genes involved in inflammation-associated pathways were shown to be downregulated in the 4°C 5-week group. Therefore, the results of the present study indicate that tissue hypoxia and increased oxidative stress may play important roles in the process of CIH.

## Introduction

Hypertension has an increased prevalence in cold regions or during winter. Cold winters increase the severity of hypertension and trigger myocardial infarction and stroke in hypertensive patients, leading to high mortality and morbidity rates from cardiovascular complications (1–6). Therefore, it is important to further investigate the mechanisms underlying cold-induced hypertension (CIH). Previous studies that have focused on clinical epidemiology in humans have indicated that cold exposure is a risk factor of hypertension (6–8). However, the cause of CIH remains unknown. The thermoregulatory-vascular remodeling hypothesis indicates that avoiding the ingestion of sodium chloride is key to preventing hypertension (9). By contrast, advances in mechanistic studies of CIH in animals indicate that the activated sympathetic nervous system (10,11) initiates CIH via the renin-angiotensin system (12,13). Additional studies have also revealed that cold exposure suppresses the expression of endothelial nitric oxide synthase and thus the formation of nitric oxide (14), as well as increasing the production of endothelin-1 (15). The hypothesis that hypertension is caused by increased oxidative stress remains controversial (16–20).

In the present study, gene expression data from cold-exposed mice, generated by an Affymetrix microarray, were selected from the Gene Expression Omnibus (GEO) database. Gene expression analyses were performed with the aim of identifying the expression patterns of CIH-associated genes. Integrating the results of the present study with previous related studies allowed a more comprehensive interpretation of the molecular mechanism underlying CIH to be constructed at the gene expression level.

## Materials and methods

### Microarray data

The GSE13432 dataset was downloaded from the GEO database (http://www.ncbi.nlm.nih.gov/geo/). Sample information of the 12 C57/Bl6 mice that were maintained at 30 or 4°C for 1 or 5 weeks is listed in Table I. Data from the three mice in each group were used for gene expression analysis using the Mouse Genome 430 2.0 Array system (Affymetrix, Inc., Santa Clara, CA, USA). The robust multichip analysis algorithm was used to polish the microarray data prior to further analysis (21).

### Differentially expressed gene (DEG) detection

Based on the Bayesian statistical model, DEGs were identified between the 4 and 30°C groups at the various timescales (4°C 1 week/30°C 1 week, 4°C 5 weeks/30°C 5 weeks). Statistical t-tests with multiple test correction using the Benjamini and Hochberg procedure (22) were performed with the threshold of significantly expressed genes set at a false discovery rate (FDR) of 0.01. Up- or downregulation of DEGs was determined as fold change.

### Gene Ontology (GO) and pathway analysis of DEGs

All cold-induced DEGs were mapped according to the GO (http://www.geneontology.org) and Kyoto Encyclopedia of Genes and Genomes pathway terms (http://www.genome.jp/kegg/). Multiple models for pathway enrichment evaluation were utilized (23,24). Blood pressure-associated DEGs were defined by blood pressure-associated GO function terms, while hypertension-associated DEGs were selected from hypertension-associated pathways identified by known hypertension genes in the Online Mendelian Inheritance in Man (OMIM) database and the Mammalian Phenotype Ontology in the Mouse Genome Informatics (MGI) database (http://www.omim.org and http://www.informatics.jax.org/). Odds ratios and FDRs were calculated with a threshold FDR value of 0.05 to identify statistical significance of pathway enrichment. Kernel principal component analysis was used to evaluate the overall gene expression differences between samples. All these procedures were performed with R statistical software (v 2.14.1; http://www.r-project.org/), and Bioconductor limma packages (3.12.1) and libraries (25) (http://www.bioconductor.org/packages/devel/bioc/html/limma.html).

### Protein-protein interaction (PPI) network analysis

Network inference was performed using Cytoscape (26) (v 2.8.3; http://www.cytoscape.org/). The interaction dataset between genes was downloaded from the Biomolecular Interaction Network Database (http://bind.ca) and the MIPS Mammalian Protein-Protein Interaction Database (MPPI; http://mips.helmholtz-muenchen.de/proj/ppi/). The network was analyzed for cold-induced DEGs associated with blood pressure regulation and hypertension in two case/control groups (4°C for 1 week vs. 30°C for 1 week and 4°C for 5 weeks vs. 30°C for 5 weeks).

## Results

### Cold-induced DEG profiling

The downloaded GSE13432 dataset contained the gene expression data of four groups of mice (three replicates in each) exposed to four conditions: 4°C for 1 and 5 weeks and their respective controls at 30°C for 1 and 5 weeks (27). In order to demonstrate the gene expression level changes that resulted from cold exposure, analysis of DEGs was performed between the cold exposure mice and the control groups for the two timescales. Multiple testing with FDR control was conducted to improve the accuracy of the results.

**Figure 1:**
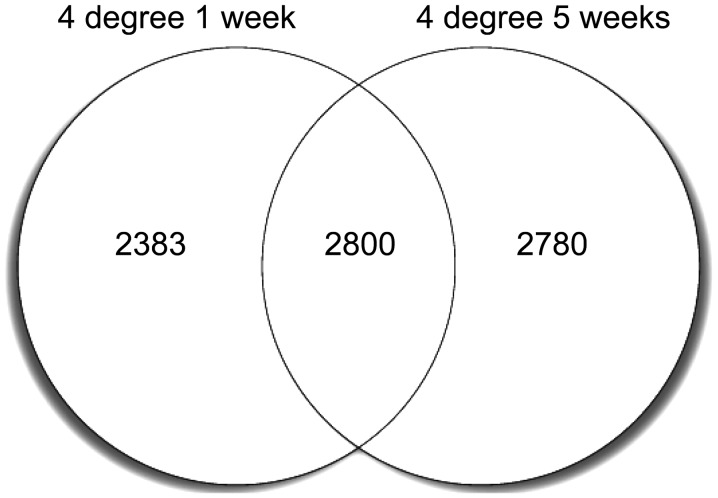
Venn plot showing the identified DEGs in the two cold-exposure groups. A total of 5,183 DEGs were identified in the 4°C 1 week vs. 30°C 1 week group, while 5,580 DEGs were identified in the 4°C 5 week vs. 30°C 5 week group (FDR, <0.01). Among the DEGs, 2,800 genes maintained increased or decreased expression levels after week 1 and 5, while >2,000 genes were detected only at week 1 or 5. DEGs, differentially expressed genes; FDR, false discovery rate.

In total, 5,183 DEGs were identified in the 4°C 1 week vs. 30°C 1 week group, while 5,580 DEGs were identified in the 4°C 5 week vs. 30°C 5 week group (FDR, <0.01). Among these DEGs, 2,800 genes maintained increased or decreased expression levels at week 1 and 5, while >2,000 genes were detected only at week 1 or 5, as shown in Fig. 1.

With regard to the most significant DEGs, the expression levels of uncoupling protein 1 (*UCP1*), a mitochondrial proton carrier, and a series of other fatty acid metabolic genes, including *ELOVL3*, *FABP3* and *CPT1B*, were markedly elevated in the two cold exposure groups. In addition, energy metabolism genes associated with oxidative phosphorylation, the citrate cycle and mitochondrial function were also shown to be upregulated, including the ubiquinol-cytochrome *c* reductase (*UQCR*) and cytochrome *c* oxidase (*COX*) gene families. Peroxisome genes were also activated in the two groups. Notably, following cold exposure, the number of downregulated genes was nearly double the number of upregulated genes.

### Cold exposure regulates the expression levels of blood pressure-associated genes

**Figure 2:**
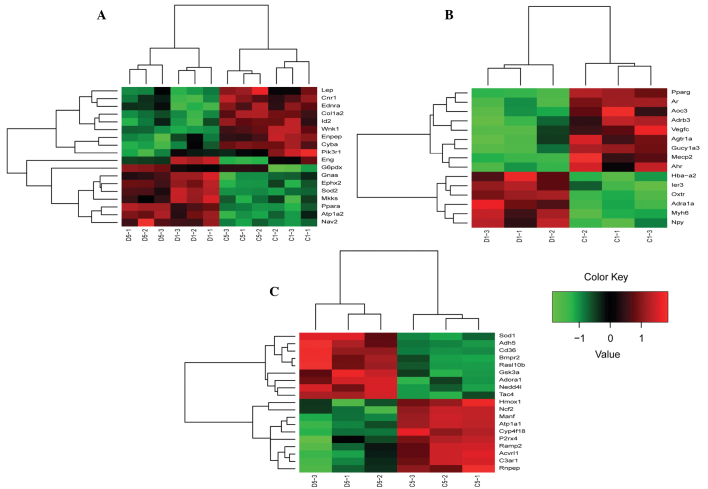
Heatmaps demonstrating the expression levels of blood pressure-associated genes in (A) all mice and the (B) 4°C 1 week and (C) 4°C 5 week groups. Information for each sample corresponding to Table I is shown on the x-axis.

A total of 52 DEGs associated with blood pressure regulation were selected according to the GO terms and descriptions. Expression patterns were constructed with clustering analysis and are shown in Fig. 2. In total, 18 DEGs were observed that were differentially expressed after 1 and 5 weeks of exposure. In addition, six genes were identified to have a larger difference at the expression level with long term exposure, including superoxide dismutase 2 (*SOD2*), glucose-6-phosphate dehydrogenase X-linked (*G6pdx*), WNK lysine deficient protein kinase 1 (*Wnk1*), peroxisome proliferator activated receptor α (*Ppara*), cytochrome b-245, α polypeptide (*Cyba*) and ATPase, Na^+^/K^+^ transporting, α 2 polypeptide (*Atp1a2*). Furthermore, there were a number of 1 week specific DEGs that exhibited a transient expression pattern following 1 week exposure, but returned to the initial values by week 5, including angiotensin II receptor type 1a (*Agtr1a*) and adrenergic receptor α 1a (*Adra1a*). There were also specific genes considered to be slow-response genes that were only observed after 5 weeks of cold exposure.

### Hypertension-associated pathway analysis

In order to exclude unassociated pathways that were caused by cold acclimation, the OMIM and MGI databases were searched and 51 genes were identified as known hypertension-associated genes. In order to identify how these genes were affected by cold exposure, enrichment analysis was performed in the associated pathways. Nervous system-associated pathways, including Huntington’s and Alzheimer’s diseases, were found to be significantly enriched (odds ratio, >2; FDR, <10^−6^) in the two cold exposure groups. In addition, immune system pathways were specifically enriched in the 5 week exposure group, including the chemokine signaling pathway, B cell receptor signaling pathway and Fc γ R-mediated phagocytosis (Table II).

### PPI network

According to the MPPI database, numerous genes (4°C 1 week group, 327; 4°C 5 week group, 515) were identified for construction of the network. It was possible to organize the majority of the genes into a single network. Proteins associated with signal transduction were observed in the PPI network of the 4°C 1 week group, including certain proteins associated with the hypoxia-inducible factor (HIF-1) and mitogen-activated protein kinase (MAPK) signaling pathways. A number of additional proteins associated with the immune system were identified in the 4°C 5-week mice, including mammalian target of rapamycin (mTOR) and B and T cell receptor signaling pathways.

## Discussion

Cold exposure is hypothesized to be an important factor contributing to hypertension. However, the pathogenic mechanism of CIH is not fully understood. Although hypertension is defined as a condition of high blood pressure, the disorder is complex with numerous phenotypes and a number of causative factors, including genetics and environmental factors (8). Thus, mice models that were only exposed to cold were selected for the present study. The results of the present study indicate that a myriad of genes were expressed differentially when adapting to cold acclimation. Genes associated with thermogenesis (*UCP1*) and energy metabolism (*UQCR* and *COX* families) were activated as more heat and energy were required to maintain temperature homeostasis (9). Thus, the compensatory increase in the metabolic rate causes tissue hypoxia, as previously demonstrated (27). The expression of endothelial PAS domain-containing protein 1 (*Epas1*) was upregulated, indicating that the HIF signaling pathway was activated. HIF-associated genes also exhibited regulated expression levels and were observed in the PPI networks. Genes associated with oxidative stress, including *SOD2* and epoxide hydrolase 2 (*EPHX2*), were also shown to be significantly upregulated with DEG and pathway analyses of blood pressure-associated genes. In addition, hypertension-associated gene analysis revealed that pathways associated with oxidative stress were markedly enriched in the initial exposure group (4°C for 1 week). Huntington’s and Alzheimer’s disease pathways are associated with mitochondrial dysfunction and the peroxisome pathway plays a crucial role in free radical detoxification. Furthermore, a number of additional immune system pathways were enriched in the chronic exposure group (4°C for 5 week), which may further induce inflammatory reactions and increase oxidative stress (28–30). Therefore, the results of the present study indicate that an imbalance in reactive oxygen species and superoxide caused by hypoxia and oxidative stress may contribute to the initiation of hypertension, while chronic cold exposure may accumulate the effect of oxidative stress in the process of hypertension.

## Figures and Tables

**Table I:** Characteristics of the sample information.

GEO identification	Sample description	Sample name	Raw file data
GSM338983	1 week 30°C rep 1	C1-1	GSM338983.cel.gz
GSM338984	1 week 30°C rep 2	C1–2	GSM338984.cel.gz
GSM338985	1 week 30°C rep 3	C1-3	GSM338985.cel.gz
GSM338986	1 week 4°C rep 1	D1-1	GSM338986.cel.gz
GSM338987	1 week 4°C rep 2	D1-2	GSM338987.cel.gz
GSM338988	1 week 4°C rep 3	D1-3	GSM338988.cel.gz
GSM338989	5 weeks 30°C rep 1	C5-1	GSM338989.cel.gz
GSM338990	5 weeks 30°C rep 2	C5-2	GSM338990.cel.gz
GSM338991	5 weeks 30°C rep 3	C5-3	GSM338991.cel.gz
GSM338992	5 weeks 4°C rep 1	D5-1	GSM338992.cel.gz
GSM338993	5 weeks 4°C rep 2	D5-2	GSM338993.cel.gz
GSM338994	5 weeks 4°C rep 3	D5-3	GSM338994.cel.gz

GEO, Gene Expression Omnibus.

**Table II:** KEGG enriched pathways of hypertension-associated genes in the OMIM and MGI databases.

Pathways	Odds ratio	P-value	FDR	Subclass
4°C 1 week group				
Huntington’s disease	3.087	7.465E-13	4.852E-11	Neurodegenerative disease
Alzheimer’s disease	2.924	1.590E-11	8.270E-10	Neurodegenerative disease
Peroxisome	2.824	3.937E-06	1.462E-04	Transport and catabolism
Fatty acid metabolism	3.635	2.425E-05	6.736E-04	Lipid metabolism
4°C 5 week group				
Huntington’s disease	2.631	6.559E-10	4.263E-08	Neurodegenerative disease
Alzheimer’s disease	2.433	1.903E-08	8.246E-07	Neurodegenerative disease
Phagosome	2.364	7.954E-08	2.954E-06	Transport and catabolism
Epstein-Barr virus infection	1.988	1.306E-06	3.525E-05	Infectious disease
Toxoplasmosis	2.483	1.746E-06	4.127E-05	Infectious disease
Chemokine signaling	1.976	4.920E-06	9.841E-05	Immune system
Osteoclast differentiation	1.989	1.207E-04	1.847E-03	Development
B cell receptor signaling	2.330	1.886E-04	2.725E-03	Immune system
Tuberculosis	1.744	2.129E-04	2.913E-03	Infectious disease
Hepatitis B	1.807	3.237E-04	4.208E-03	Infectious disease
Viral carcinogenesis	1.618	4.905E-04	5.545E-03	Cancer
Fc γ R-mediated phagocytosis	2.038	7.372E-04	7.986E-03	Immune system
Leishmaniasis	2.108	1.980E-03	1.716E-02	Infectious disease
Pertussis	2.013	2.506E-03	1.975E-02	Infectious disease
Acute myeloid leukemia	2.112	3.787E-03	2.825E-02	Cancer
Hepatitis C	1.605	5.119E-03	3.597E-02	Infectious disease
Peroxisome	1.825	5.296E-03	3.624E-02	Transport and catabolism
Estrogen signaling	1.723	5.638E-03	3.758E-02	Endocrine system
Fatty acid metabolism	2.168	8.170E-03	4.968E-02	Lipid metabolism
T cell receptor signaling	1.623	8.245E-03	4.968E-02	Immune system
Leukocyte transendothelial migration	1.597	8.364E-03	4.968E-02	Immune system

KEGG, Kyoto Encyclopedia of Genes and Genomes ; OMIM, Online Mendelian Inheritance in Man; MGI, Mouse Genome Informatics; FDR, false discovery rate.
